# Cushing's syndrome is associated with an advanced chronotype

**DOI:** 10.1111/jne.70286

**Published:** 2026-10-08

**Authors:** Hanna Nowotny, Adriana Albani, Mina Mazari, Stephanie Zopp, Martha Merrow, Nicole Reisch, Luis Gustavo Perez‐Rivas, Marily Theodoropoulou

**Affiliations:** ^1^ Medizinische Klinik und Poliklinik IV, LMU Klinikum, LMU Medizin Ludwig‐Maximilians‐Universität München Munich Germany; ^2^ Institute of Medical Psychology, LMU Medizin Ludwig‐Maximilians‐Universität München Munich Germany

**Keywords:** actigraphy, chronotype, circadian rhythm, Cushing's syndrome, Munich chronotype questionnaire

## Abstract

Cushing's syndrome is characterised by chronic glucocorticoid excess that abolishes the physiological circadian cortisol rhythm. We investigated whether this circadian disruption alters chronotype. In a single‐centre case–control study, chronotype was assessed using the Munich ChronoType Questionnaire (MCTQ; *n* = 119) and wrist actigraphy (*n* = 39) in patients with active Cushing's syndrome, patients in biochemical remission, and healthy controls. The primary outcome was mid‐sleep on free days corrected for sleep debt (MSFsc). Multivariable analysis was performed to identify independent predictors of chronotype. Agreement between methods was assessed by Bland–Altman analysis. Patients with active Cushing's syndrome had a significantly earlier chronotype than patients in remission and controls (MSFsc by MCTQ: 2.38 h [IQR 1.22; 3.41] vs. 3.29 h [IQR 2.65; 4.32] vs. 3.73 h [IQR 3.16; 4.08]; *p* = .007 and *p* = .0003, respectively; actigraphy: 3.15 ± 0.90 h vs. 4.03 ± 0.96 h vs. 4.46 ± 0.75 h; *p* = .039 and *p* = .001, respectively). Chronotype did not differ significantly between patients in remission and controls. In a multivariable quantile regression model, active Cushing's syndrome was an independent predictor of chronotype (1.32 h earlier than controls; *p* = .049), while age, BMI, psychiatric symptoms and sleep disturbance had no significant influence. Actigraphy confirmed earlier sleep offset and acrophase in active Cushing's syndrome. MCTQ and actigraphy were significantly correlated (Pearson *r* = 0.619, *p* = .0003, *n* = 29); agreement analysis revealed no systematic bias, but a significant proportional bias, reflecting wider dispersion of MCTQ‐derived values. Patients with active Cushing's syndrome have an earlier chronotype than patients in remission and healthy controls, as determined by both subjective and objective methods. Chronotype assessment, particularly with the MCTQ, may complement biochemical monitoring.

## 1 INTRODUCTION

Endogenous Cushing's syndrome is an endocrine disorder characterised by chronic cortisol excess causing severe comorbidities and high mortality.^1^, ^2^ The most frequent cause of endogenous cortisol excess is ACTH‐producing pituitary tumours (Cushing's disease) followed by adrenal adenomas. In very rare cases, Cushing's syndrome is due to extra‐pituitary (ectopic) ACTH‐secreting neuroendocrine tumours, or to adrenocortical cancers.^3^ Patients suffer from metabolic syndrome, hypertension, osteoporosis, myopathy, reproductive comorbidities, immune dysregulation, collectively resulting in increased morbidity and mortality.^4^, ^5^ Patients may also present psychiatric symptoms that include depression and anxiety, and sleep disturbances.^6^, ^7^, ^8^

Cortisol secretion follows a distinct circadian rhythm, with an early morning maximum, decreasing levels during the day, a nadir at around midnight and an abrupt elevation in late sleep peaking again shortly after awakening (cortisol awakening response, CAR).^9^ In patients with Cushing's syndrome, regardless of aetiology, this circadian modulation is lost and cortisol concentrations follow a flattened rhythm that remains elevated during the night.^10^ Glucocorticoid itself is a zeitgeber of the circadian clock at the cellular level, thus a reorganisation of the circadian system would be predicted in patients with Cushing's syndrome.^11^

The circadian clock reveals itself in behaviour through the phase or timing of the sleep/wake cycle, called chronotype, and this timing is mediated at the level of the suprachiasmatic nucleus (SCN). Chronotype is quantified as an individual's preferred timing of sleep and wakefulness under socially unconstrained conditions. It does not reflect the quality of sleep or its architecture, but individual differences in the circadian clock and its entrainment.^12^ Chronotype is affected by age (adolescents have later chronotype), but also by lifestyle factors like smoking and night shift work.^13^, ^14^ Although a clear association between chronotype and cortisol concentrations has been difficult to establish, there is evidence that cortisol levels are higher after awakening in subjects with an earlier chronotype.^15^, ^16^, ^17^, ^18^ At issue is whether functional glucocorticoid receptors are expressed in the SCN or not, an obvious prerequisite for a direct link between glucocorticoid levels and circadian clock‐regulated behaviour.^19^, ^20^ In that sense, the cohort of Cushing's syndrome patients is a fascinating group of individuals to study regarding the timing of daily behaviour.

Whether cortisol excess alters chronotype has not been systematically investigated. A short‐term study evaluating actigraphy data obtained during a 3‐day period reported higher fragmented sleep and increased nocturnal motor activity in patients with Cushing's syndrome.^21^ In the present study, we assessed chronotype in patients with active Cushing's syndrome or in remission, utilising both subjective (Munich Chronotype Questionnaire) and objective (actigraphy) methods. MCTQ collects data on sleep and activity times separately for work and work‐free days. This allows the estimation of chronotype as mid‐sleep time on free days (MSF; the half‐way point between sleep onset and sleep end) corrected for the sleep debt accumulated during the work week (MSFsc).^12^ In actigraphy, wrist wearable devices record motor activity (actimetry) and provide data on rest‐activity over long periods of time that can be used to monitor movement activity and sleep–wake cycles.^22^ Using both methods, we determined and compared chronotype between patients with active Cushing's syndrome, patients with previous Cushing's syndrome being in remission at the time of the study, as well as healthy controls with no history or evidence of Cushing's syndrome.

## 2 METHODS

### 2.1 Study design

Single‐centre study conducted at the Department of Medicine IV of the LMU University Hospital, between September 2019 and May 2024 (Ethics approval No. 19‐0390; LMU University Hospital Ethics Committee). All participants provided written informed consent. Three groups were enrolled: (i) patients with confirmed, untreated active endogenous Cushing's syndrome (Cushing's disease, adrenal, or ectopic); (ii) patients diagnosed with Cushing's syndrome who were in biochemical remission at the time of assessment; and (iii) control group participants without a history of Cushing's syndrome. Remission was defined by 8:00 am serum cortisol <5 μg/dL requiring glucocorticoid replacement therapy, or by normal cortisol and normal postoperative UFC and cortisol suppression after low‐dose dexamethasone test. Clinical characteristics were collected retrospectively and are summarised in Table 1 (MCTQ cohort) and Table 2 (Actigraphy cohort).

**TABLE 1:** Clinical characteristics of the MCTQ cohort (*n* = 119).

	Total	Active disease	Remission	Control	*p*‐Value
Age, median (IQR), *n* = 119	42.5 (34.8; 54.3)	37.3 (29.6; 52.9)	47.4 (38.7; 55.3)	39.5 (34.5; 54.3)	.242
Female, *n*/*N* (%)	93/119 (78.2)	21/24 (87.5)	45/61 (73.8)	27/34 (79.4)	.391
BMI, median (IQR), *n* = 119	26.7 (22.8; 30.7)	29 (26.2; 32.6)	26.5 (22.8; 30.6)	25.3 (20.7; 29.6)	.084
BMI <25	48/119 (40.3)	5/24 (20.8)	26/61 (42.6)	17/34 (50)	.180
BMI 25–29.9	36/119 (30.3)	10/24 (41.7)	16/61 (26.2)	10/34 (29.4)	
BMI ≥30	35/119 (29.4)	9/24 (37.5)	19/61 (31.1)	7/34 (20.6)	
Menopause, *n*/*N* (%)	30/83 (36.1)	6/20 (30.0)	20/44 (45.5)	4/19 (21.1)	.153
Smoker, *n*/*N* (%)	15/119 (12.6)	9/24 (37.5)	6/61 (9.8)	0/34 (0)	<.001
Disease subtype					
Cushing's disease, *n*/*N* (%)	62/85 (72.9)	15/24 (62.5)	47/61 (77.0)	‐	.290
Adrenal, *n*/*N* (%)	19/85 (22.4)	8/24 (33.3)	11/61 (18.0)	‐	
Ectopic, *n/N* (%)	4/85 (4.7)	1/24 (4.2)	3/61 (4.9)	‐	
Comorbidities					
Diabetes, *n*/*N* (%)	10/99 (10.1)	3/24 (12.5)	5/61 (8.2)	2/14 (14.3)	.701
Metabolic syndrome, *n*/*N* (%)	29/99 (29.3)	10/24 (41.7)	15/61 (24.6)	4/14 (28.6)	.284
Hypercholesterolemia, *n*/*N* (%)	39/99 (39.4)	16/24 (66.7)	18/61 (29.5)	5/14 (35.7)	.007
Hypertriglyceridemia, *n*/*N* (%)	22/99 (22.2)	8/24 (33.3)	10/61 (16.4)	4/14 (28.6)	.190
Mixed hyperlipidaemia, *n/N* (%)	15/99 (15.2)	7/24 (29.2)	7/61 (11.5)	1/14 (7.1)	.095
Hypertension, *n/N* (%)	45/99 (45.5)	16/24 (66.7)	22/61 (36.1)	7/14 (50)	.038
Arteriosclerosis, *n/N* (%)	4/99 (4)	1/24 (4.2)	2/61 (3.3)	1/14 (7.1)	.771
CV events, *n/N* (%)	6/99 (6.1)	1/24 (4.2)	3/61 (4.9)	2/14 (14.3)	.389
Sleep apnoea, *n*/*N* (%)	5/99 (5.1)	0/24 (0)	4/61 (6.6)	1/14 (7.1)	.517
Psychiatric disorders, *n*/*N* (%)	28/99 (28.3)	9/24 (37.5)	17/61 (27.9)	2/14 (14.3)	.326
Sleep disturbance, *n*/*N* (%)	30/99 (30.3)	14/24 (58.3)	11/61 (18)	5/14 (35.7)	.001

*Note*: Kruskal–Wallis test was used for continuous variables. Chi‐square test was used for categorical variables. Fisher's exact test was used for categorical variables when cases were *n* < 5 for any category.

Abbreviations: BMI, body mass index; MCTQ, Munich chronotype questionnaire.

**TABLE 2:** Clinical characteristics of the actigraphy cohort (*n* = 39).

	Total	Active disease	Remission	Control	*p*‐Value
Age, median (IQR), *n* = 39	35.8 (29.9; 55.5)	35.6 (31.0; 57.3)	39.7 (25.2; 64.56)	35.6 (29.8; 49.6)	.917
Female, *n*/*N* (%)	33/39 (84.6)	13/13 (100.0)	11/13 (84.6)	9/13 (69.2)	.135
BMI, mean (SD), *n* = 39	26.1 (5.1)	27.6 (4.6)	27.6 (5.4)	23.1 (4.3)	.031
BMI <25	19/39 (48.7)	4/13 (30.8)	5/13 (38.5)	10/13 (76.9)	.223
BMI 25–29.9	12/39 (30.8)	6/13 (46.2)	4/13 (30.8)	2/13 (15.4)	
BMI ≥30	8/39 (20.5)	3/13 (23.1)	4/13 (30.8)	1/13 (7.7)	
Menopause, *n*/*N* (%)	8/27 (29.6)	4/12 (33.3)	3/11 (27.3)	1/4 (25%)	1.000
Smoker, *n*/*N* (%)	7/39 (17.9)	4/13 (30.8)	0/13 (0.0)	3/13 (23.1)	.128
Disease subtype					.673
Cushing's disease, *n*/*N* (%)	18/26 (69.2)	8/13 (61.5)	10/13 (76.9)	‐	
Adrenal, *n*/*N* (%)	8/26 (30.8)	5/13 (38.5)	3/13 (23.1)	‐	
Ectopic, *n*/*N* (%)	0/26 (0.0)	0/13 (0.0)	0/13 (0.0)	‐	
Comorbidities					
Diabetes, *n*/*N* (%)	2/31 (6.5)	0/13 (0.0)	2/13 (15.4)	0/5 (0.0)	.637
Metabolic syndrome, *n*/*N* (%)	6/30 (20)	4/13 (30.8)	2/13 (15.4)	0/4 (0.0)	.562
Hypercholesterolemia, *n*/*N* (%)	12/30 (40)	9/13 (69.2)	3/13 (23.1)	0/4 (0.0)	.012
Hypertriglyceridemia, *n*/*N* (%)	6/30 (20)	3/13 (23.1)	3/13 (23.1)	0/4 (0.0)	.699
Mixed hyperlipidaemia, *n*/*N* (%)	5/30 (16.7)	3/13 (23.1)	2/13 (15.4)	0/4 (0.0)	.829
Hypertension, *n*/*N* (%)	14/31 (45.2)	7/13 (53.8)	6/13 (46.2)	1/5 (20.0)	.556
Arteriosclerosis, *n*/*N* (%)	1/31 (3.2)	0/13 (0.0)	0/13 (0.0)	1/5 (20.0)	.161
CV events, *n*/*N* (%)	3/31 (9.7)	1/13 (7.7)	0/13 (0.0)	2/5 (40.0)	.060
Sleep apnoea, *n*/*N* (%)	1/31 (3.2)	0/13 (0.0)	0/13 (0.0)	1/5 (20.0)	.161
Psychiatric disorders, *n*/*N* (%)	7/30 (23.3)	5/13 (38.5)	2/13 (15.4)	0/4 (0.0)	.288
Sleep disturbance, *n*/*N* (%)^a^	9/30 (30.0)	8/13 (61.5)	1/13 (7.7)	0/4 (0.0)	.007

*Note*: ANOVA test was used to test means in continuous variables. Kruskal–Wallis test was used for continuous variables. Chi‐square test was used for categorical variables. Fisher's exact test was used for categorical variables when cases were *n* < 5 for any category.

Abbreviation: BMI, body mass index.

^a^ Patients were not under treatment for sleep disturbance.

Exclusion criteria comprised shift work, a 7‐day working week, absence of an alarm‐free free day, unclear work/free day distinctions, use of sleep medication, and (in the case of active Cushing's syndrome) the use of cortisol‐suppressing medication.

### 2.2 Munich ChronoType questionnaire

A total of 119 participants completed the MCTQ (English version 2015‐01): 24 active Cushing's syndrome, 61 remission, and 34 controls (MCTQ cohort). The questionnaire captures bedtime, sleep onset (SO = SPrep + SLat, where SPrep is the local time of preparing to sleep and SLat is the sleep latency), sleep end (SE), and sleep duration (SD = SE − SO) separately for workdays (WD) and free days (FD). Mid‐sleep time on free days (MSF) was calculated as MSF = SO_f_ + SD_f_/2. Average weekly sleep duration was derived as SD_week_ = (SD_w_ × WD + SD_f_ × FD) / 7. The primary chronotype metric, MSFsc, was computed as MSFsc = MSF − (SD_f_ − SD_week_)/2.

The standard MCTQ algorithm applies MSFsc only when SD_f_ exceeds SD_w_, using uncorrected MSF otherwise. In this study, MSFsc was applied uniformly to all participants because endogenous hypercortisolism was expected to disrupt normal workday‐free‐day asymmetry in sleep duration independently of social constraints. MSF is reported as a confirmatory secondary metric to avoid potential misleading of sleep‐debt correction. Eight non‐working participants were assigned free‐day values for all days.

### 2.3 Actigraphy

A set of 39 participants (13 per group) wore a MotionWatch 8 (CamNtech, Cambridge, UK; RRID: SCR_016643) on the non‐dominant wrist for a minimum of 14 days (60‐s epochs, MotionWatch Mode 1). From these, 29 also completed the MCTQ (7 controls, 12 patients with active Cushing's syndrome and 10 patients in biochemical remission).

Raw activity and light‐count data were exported using MotionWare software (CamNtech, Version 1.1.25.0) and stratified by WD (Sunday 12:00 to Friday 11:59) and FD (Friday 12:00 to Sunday 11:59). Sleep onset and offset were determined from immobility‐based criteria using ClockLab Analysis software (Actimetrics, Wilmette, IL, USA, Version 6.1.14). The acrophase of activity (peak daytime activity hour or peak phase), diurnal activity count (alpha counts) and nocturnal activity count (rho counts) were derived from the ClockLab activity profile. In brief, alpha counts refer to the activity counts falling into the behavioural daytime. This is not necessarily equivalent to the light versus the dark phase, but to the portion of the day when the subject is active. Rho counts refer to the activity counts falling during the inactive (or less active) part of the day, presumed to be associated with sleep. A low alpha to rho ratio would suggest more nighttime activity or sleep disturbance. Acrophase is calculated as the centre of mass of activity over the day. In our study, all of these calculations were performed within the ClockLab algorithm.

### 2.4 Statistical analysis

Statistical analysis was performed in SPSS version 30.0.0.0 (IBM). Normal distribution of continuous data was assessed by the use of histograms, quantile–quantile plots, presence of outliers, and the Shapiro–Wilk test. Variables following normal distribution were summarised as means (SD), which were compared among groups using Student's *t*‐test or one‐way ANOVA. Non‐normally distributed data were summarised as median (first quartile; third quartile) and compared with Mann–Whitney *U* and Kruskal–Wallis tests. Multivariable analysis was conducted with multiple quantile regression. *p*‐values were adjusted for multiple comparisons using Bonferroni or Tukey's correction. Bivariate associations between continuous variables were determined by Pearson's correlation or Spearman's rho. Categorical variables were analysed using chi‐square and Fisher's exact tests. Agreement between MCTQ and actigraphy data was assessed by Bland–Altman analysis, and reported as the mean difference between MCTQ and actigraphy values. Precision was reported as the limits of agreement. Proportional data were analysed by correlation of differences versus means of MCTQ and actigraphy data. Figure 1 was plotted in R version 4.5.2 using package ggplot2. Kernel density estimate plots were calculated with reduced bandwidth (smooth reduction) to avoid over‐smoothing of sample distribution. *p*‐values <.05 were considered statistically significant.

**FIGURE 1:**
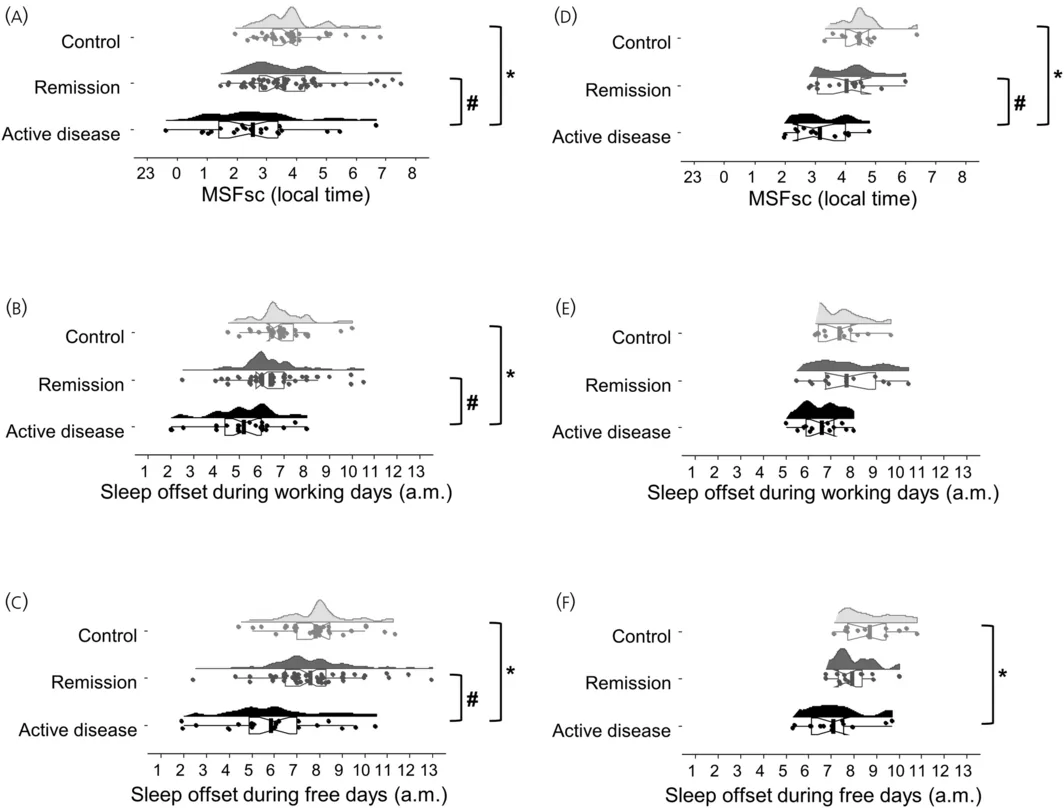
Density plots and notched boxplots depicting chronotype (MSFsc) and sleep offset on workdays and work‐free days in active Cushing's syndrome versus remission versus controls as determined by MCTQ and actigraphy. (A–C) MCTQ cohort (active Cushing's syndrome, *n* = 24, black; remission, *n* = 61, grey; controls *n* = 34, light gray). (A) MSFsc (*p = .0003 active vs. controls, #*p* = .007 active vs. remission). (B) Sleep offset on workdays (WD; *p < .0001 active vs. controls, #*p* = .007 active vs. remission). (C) Sleep offset on work‐free days (FD; *p = .0003 active vs. controls, #*p* = .020 active vs. remission). Density plots show sample distribution. Notched boxplots show IQR (box), mean (bar) and median (notch). Groups were compared using Kruskal–Wallis test adjusted for multiple comparisons using Dunn's method with Bonferroni correction. (D–F) Actigraphy cohort (active *n* = 13, remission *n* = 13, and controls *n* = 13). (D) MSFsc (*p = .001 active vs. controls, #*p* = .039 active vs. remission). (E) Sleep offset on workdays (WD). No significant differences were recorded between any of the groups. (F) Sleep offset on work‐free days (FD) (*p = .003 active vs. controls). Density plots show sample distribution. Notched boxplots show IQR (box), mean (bar) and median (notch). Groups were compared using ANOVA corrected with Tukey's adjustment for multiple comparisons.

## 3 RESULTS

### 3.1 Cohorts

The MCTQ cohort included 119 participants: 24 patients with active Cushing's syndrome (21 female, median age at participation 37.3 years [IQR 29.6; 52.9]), 61 patients in remission (45 female, 47.4 years [IQR 38.7; 55.3]), and 34 control participants (27 female, 39.5 years [IQR 34.5; 54.3]) (Table 1). The actigraphy cohort included 39 participants: 13 with active Cushing's syndrome (13 female, 35.6 years [IQR 31.0; 57.3]), 13 in remission (11 female, 39.7 years [IQR 25.2; 64.56]), and 13 controls (9 female, 35.6 years [IQR 29.8; 49.6]) (Table 2).

The majority of patients had Cushing's disease, followed by adrenal Cushing's (Tables 1 and 2). The MCTQ cohort also included one patient with active Cushing's syndrome and three in remission due to ectopic ACTH‐producing neuroendocrine tumour.

Median remission duration was 48.3 months (IQR 24.4–120.5) for the MCTQ cohort and 46.7 months (IQR 24.3–83.5) for the actigraphy cohort. Thirty and six patients in remission from the MCTQ and actigraphy cohorts respectively received glucocorticoid substitution therapy (median hydrocortisone dose 20 and 25 mg/day, respectively) at the time of enrolment.

### 3.2 Chronotype

#### 3.2.1 MCTQ

Patients with active Cushing's syndrome exhibited a significantly earlier chronotype compared to both patients in remission and controls (MSFsc: 2.38 h [IQR 1.22; 3.41] vs. 3.29 [IQR 2.65; 4.32], and vs. 3.73 h [IQR 3.16; 4.08]; *p* = .007 and *p* = .0003 respectively; Table 3, Figure 1A). The chronotype of patients in remission did not differ significantly from that of controls (*p* = .456). Similar results were obtained with MSF (Table 3).

**TABLE 3:** Chronotype in patients with active Cushing's syndrome versus remission versus controls as determined by MCTQ and actigraphy.

	Active disease	Remission	Controls	*p*‐Value active versus control	*p*‐Value active versus remission	*p*‐Value remission versus control
MCTQ^a^ Median (IQR)	*n* = 24	*n* = 61	*n* = 34			
MSFsc	2.38 (1.22; 3.41)	3.29 (2.65; 4.32)	3.73 (3.16; 4.08)	.0003	.007	.456
MSF	2.61 (1.47; 3.45)	3.58 (2.88; 4.35)	3.88 (3.48; 4.22)	.001	.004	.972
Weekly sleep duration	6.16 (5.2; 6.97)	7.4 (6.57; 8.14)	7.28 (6.48; 7.82)	.036	.001	1
Sleep onset WD	23.09 (22.58; 23.94)	23.17 (22.54; 24.09)	23.65 (23.07; 24.17)	.131	1	.155
Sleep offset WD	5.25 (4.13; 6)	6 (5.67; 7)	6.54 (6.31; 7.5)	<.0001	.007	.21
Sleep duration WD	6 (5.37; 7)	7.33 (6.33; 7.92)	7.09 (6.63; 7.62)	.018	.001	1
Sleep onset FD	23.33 (22.73; 24)	23.88 (23.17; 24.52)	24.13 (23.48; 24.63)	.026	.282	.768
Sleep offset FD	6 (4.63; 7)	7.5 (6.5; 8.38)	8 (7; 8.5)	.0003	.02	1
Sleep duration FD	6.17 (5.02; 7.25)	7.83 (6.75; 8.96)	7.79 (6.88; 8.28)	.013	.02	1
Actigraphy^b^ Mean (SD)	*n* = 13	*n* = 13	*n* = 13			
MSFsc	3.15 (0.9)	4.03 (0.96)	4.46 (0.75)	.001	.039	.416
MSF	3.16 (0.95)	3.95 (0.98)	4.63 (0.85)	.0008	.09	.17
Weekly sleep duration	7.84 (0.74)	8.05 (0.75)	7.83 (0.78)	1	.749	.74
Sleep onset WD	22.63 (0.81)	23.57 (1.75)	23.66 (0.95)	.1	.149	.977
Sleep offset WD	6.56 (0.9)	7.67 (1.51)	7.37 (1.02)	.198	.052	.783
Sleep duration WD	7.83 (0.71)	8.11 (0.92)	7.7 (0.78)	.922	.644	.413
Sleep onset FD	23.23 (0.91)	24 (1.16)	24.55 (0.91)	.005	.136	.353
Sleep offset FD	7.1 (1.36)	7.91 (0.93)	8.71 (1.18)	.003	.191	.206
Sleep duration FD	7.86 (1.31)	7.91 (0.76)	8.16 (1.23)	.787	.994	.845

Abbreviations: FD, (work) free days; MCTQ, Munich chronotype questionnaire; MSF, mid‐sleep on free days; MSFsc, mid‐sleep on free days corrected for sleep debt; WD, workdays.

^a^ Kruskal–Wallis test corrected for multiple comparisons.

^b^ ANOVA corrected using Tukey's adjustment for multiple comparisons. MSF and MSFsc data are expressed in decimal time.

Patients with Cushing's syndrome had earlier sleep offset, that is, woke up earlier, on both work and work‐free days, compared to controls and patients in remission (Table 3; Figure 1B,C). Sleep onset did not differ significantly between the different groups on working days, but was significantly earlier for patients with Cushing's syndrome on work‐free days compared to controls but not to patients in remission. Weekly sleep duration, and sleep duration on workdays and work‐free days was significantly shorter in patients with Cushing's syndrome compared to both patients in remission and controls, while no significant differences were found between remission and controls (Table 3).

#### 3.2.2 Actigraphy

Similar to MCTQ, actigraphy data revealed significantly earlier chronotype (MSFsc) in patients with active Cushing's syndrome compared to controls and patients in remission, and no significant differences between remission and control groups (Table 3; Figure 1D). Patients with active Cushing's syndrome also had significantly earlier sleep onset and offset on work‐free days (but not on workdays) than controls (*p* = .005 and *p* = .003, respectively; Table 3; Figure 1E,F). Contrary to sleep duration data obtained from MCTQ, sleep duration measured by actigraphy did not differ significantly between the three groups (Table 3).

The acrophase (i.e., activity peak) on work‐free days was significantly earlier in patients with active Cushing's syndrome than in controls (*p* = .028; Table S1, Supporting Information). There was also a trend for higher rho counts on both work‐free days and workdays in these patients, which did not reach statistical significance (overall *p* = .054 and *p* = .088; Table S1).

### 3.3 Chronotype and clinical characteristics

In a univariable analysis, most patients' characteristics did not influence chronotype, with the exception of psychiatric symptoms (depression, explicitly mentioned change of mood) and sleep disturbances, which were associated with an earlier chronotype (MCTQ *p* = .014 and *p* = .016, respectively; Table S2).

In a multiple quantile regression analysis, we still observed earlier chronotype in patients with Cushing's syndrome (MSFsc 1.32 h earlier than controls; *p* = .049; Table 4) after adjustment for age, BMI, disease status, psychiatric symptoms and sleep disturbance. We also conducted a subgroup analysis restricted to non‐smoking participants as a sensitivity analysis (total *n* = 104; with clinical information *n* = 84; Tables S3 and S4) to address the effect of smoking as a potential confounder. In this restricted subgroup, multivariable analysis showed that participants with active disease remained associated with an earlier chronotype (MSFsc 1.48 h earlier than controls; *p* = .044, Table 4), indicating that smoking status was not a confounder variable. Age, BMI, and psychiatric symptoms were not independently associated with chronotype, whereas sleep disturbance tended to associate with an earlier chronotype in the non‐smoking subgroup (*p* = .062), but not in the complete cohort (*p* = .324).

**TABLE 4:** Multiple quantile regression analysis of MSFsc data as determined by MCTQ.

Parameter	All (*n* = 99)	*p*‐Value	Non‐smokers only (*n* = 84)	*p*‐Value
	Effect on median MSFsc (95%CI), h		Effect on median MSFsc (95%CI), h	
Status				
Active disease versus control	−1.32 (−2.63; −0.02)	.049	−1.48 (−2.91; −0.06)	.044
Remission versus control	−0.52 (−1.63; 0.59)	.359	−0.13 (−1.35; 1.08)	.831
Age	−0.03 (−0.05; 0.01)	.286	−0.02 (−0.06; 0.01)	.330
BMI	−0.01 (−0.07; 0.08)	.854	0.01 (−0.08; 0.09)	.803
Psychiatric symptoms	−0.34 (−1.04; 0.36)	.345	−0.54 (−1.25; 0.17)	.138
Sleep disturbance	−0.36 (−1.07; 0.35)	.324	−0.76 (−1.55; 0.03)	.062

Abbreviations: MCTQ, Munich chronotype questionnaire; MSFsc, mid‐sleep on free days corrected for sleep debt.

We did not perform multivariate analysis on the actigraphy cohort due to the small number of participants per group and incomplete data (Table 2).

Glucocorticoid substitution in patients in remission (*n* = 30 MCTQ; *n* = 6 actigraphy) did not significantly affect chronotype (Table S5). In the actigraphy sub‐cohort (*n* = 13), patients with glucocorticoid substitution (*n* = 6) showed significantly earlier sleep onset and sleep offset on workdays (*p* = .038 for both). However, given the small group sizes (*n* = 6 vs. *n* = 7), these findings should be interpreted with caution.

We also explored a possible relationship between chronotype changes and the degree of hypercortisolism, assessed by 24 h urinary free cortisol, late‐night salivary cortisol, and serum cortisol after a low‐dose dexamethasone suppression test (DST). We observed weak negative correlations between MSFsc measured by the MCTQ and urinary free cortisol (Spearman's rho −0.241, *p* = .028), late‐night salivary cortisol (Spearman's rho −0.309, *p* = .004) and cortisol levels after dexamethasone suppression test (Spearman's rho −0.372, *p* = .008). In contrast, when measuring chronotype using actigraphy, we found no significant correlation (Spearman's rho −0.310, −0.345 and −0.185; *p* values .150, .107 and .491, for 24 h urinary free cortisol, late‐night salivary cortisol, and cortisol after dexamethasone suppression test). In addition, we could not detect any correlation within the group of patients with active disease between hormone levels and MSFsc, regardless of the method used to assess the chronotype.

### 3.4 Agreement between MCTQ and actigraphy

Chronotype metrics from both MCTQ and actigraphy were obtained from 29 patients. MCTQ data and actigraphy measurements were significantly correlated (Table S6). Bland–Altman analysis showed no systematic bias between the two methods, but we observed positive proportional bias for MSFsc, weekly sleep duration, sleep duration during working days, and all metrics for free days (Table S6). This indicated discordance between the two methods, which was due to the higher variance of MCTQ data.

## 4 DISCUSSION

In the present study we report chronotype in patients with active endogenous Cushing's syndrome assessed with objective (actigraphy) and subjective (MCTQ) methods, compared to controls and patients in remission. We show that patients with active Cushing's syndrome, but not patients in remission, have an earlier chronotype. Furthermore, we demonstrate a stronger influence of active Cushing's syndrome on chronotype than any hypercortisolism‐related comorbidity. From the chronobiological perspective, this observation invokes Aschoff's Rule.^23^, ^24^ This formalism demonstrates that constitutive rather than cycling exposure to zeitgebers can systematically change the free running period of a circadian rhythm. For instance, nocturnal animals generally show increasing period lengths whilst diurnal animals show decreasing period lengths with increasing light levels. Period length corresponds to entrained phase as predicted by oscillator theory.^24^ By extension, the constitutive presence of zeitgebers normally expressed during the active phase, such as glucocorticoid, would be predicted to shorten the circadian period and correspondingly lead to an earlier phase of entrainment.

From the perspective of our subjects, to our knowledge, this is the first report that uses both MCTQ and actigraphy data to determine and compare chronotype in patients with active endogenous hypercortisolism, those in remission, and controls. A previous actigraphy study analysed data recorded over a 3‐day period and showed higher nocturnal activity and fragmented sleep in patients with Cushing's disease compared to healthy controls.^21^ Our actigraphy data obtained from a longer recording time (~2 weeks) supports this observation, with rho counts (indicating nocturnal activity) showing a trend towards higher levels in patients with Cushing's syndrome relative to controls, suggesting greater nocturnal restlessness in patients with active disease. Furthermore, actigraphy‐based acrophase on work‐free days (a proxy for chronotype; 22) was earlier in patients with active Cushing's syndrome than in controls. No differences were recorded on days with imposed structure (workdays).

These observations are not surprising, since Cushing's patients frequently complain of poor sleep quality, and sleep impairments have been recently reported in patients with Cushing's syndrome and mild autonomous cortisol secretion.^8^, ^25^ Cortisol and the HPA axis have been closely interconnected with sleep not only in pathological but also under physiological conditions (reviewed in ref. 26). In the general population, delayed sleep onset correlates with delayed night cortisol release, and in older (>50 years) adults the increase in evening cortisol levels was accompanied by longer daytime sleep latency and fragmented sleep.^27^, ^28^, ^29^

Cortisol secretion and circadian rhythmicity have a close reciprocal interconnection.^9^, ^30^ Patients with Cushing's syndrome show complete loss of rhythmicity in the expression of circulating circadian clock genes, which was restored for most genes after remission.^31^, ^32^ Our data indicate a similar process with chronotype, which shifts earlier in patients with Cushing's syndrome than in controls, but not in patients in remission, suggesting that constitutive and high levels of cortisol push chronotype to an earlier phase.

Prior reports have linked nicotine use to a later chronotype.^14^ In our cohort, smoking was not associated with a later phenotype assessed by MCTQ or actigraphy. Moreover, the shift in chronotype observed in patients with active disease in the whole cohort was comparable to the one observed in the restricted subgroup of non‐smoking participants. Whether cortisol excess modifies the relationship between smoking and circadian timing warrants dedicated investigation.

Using objective (actigraphy) and subjective (MCTQ) methods allowed us to take a more robust approach to chronotype determination. Both methods demonstrated an earlier chronotype (expressed as MSFsc) in patients with Cushing's syndrome than in controls and patients in remission. However, they also revealed an interesting discordance that is related to the self‐perception of sleep duration: according to MCTQ data, patients with active Cushing's syndrome had significantly shorter sleep duration than controls or patients in remission. However, objective actigraphy measures did not show significant differences in weekly sleep duration or in sleep duration in workdays and work‐free days. Agreement analysis revealed a proportional bias caused by wider dispersion of variables obtained with MCTQ than those recorded using actigraphy. These differences were observed in all groups, but they were more remarkable in the group of patients with active Cushing's syndrome. This implies that, in our study cohort, patients with active Cushing's syndrome underestimated their sleep duration and had the perception of sleeping less than they actually did.

Study limitations include a small number of participants in the actigraphy cohort, due to the limited availability of wrist wearable devices; imbalanced groups in terms of sex, BMI, smoking habits, and comorbidities (hypercholesterolemia, hypertension, sleep disturbance); and incomplete clinical data on comorbidities related to hypercortisolism, especially in the control group of the actigraphy cohort. Additional limitations are the lack of sufficient data regarding menstrual cycle phase, childcare responsibilities, and social engagement, which may have significant consequences on the chronotype. As we did not perform a longitudinal follow‐up study, we could not determine potential changes in chronotype during active disease and after biochemical normalisation in the same patients. Accordingly, our data should be interpreted with caution.

In conclusion, data derived from two independent methods revealed a shift in chronotype in patients with active Cushing's syndrome. Studies on patients with recurrence will show whether monitoring a patient's chronotype could enable the timely detection of disease recurrence, particularly when frequent biochemical monitoring is not easily accessible or feasible.

## AUTHOR CONTRIBUTIONS

**Hanna Nowotny:** Investigation; writing – original draft; methodology; software; writing – review and editing; funding acquisition. **Adriana Albani:** Conceptualization; methodology; data curation; software; investigation; project administration; resources; writing – review and editing; funding acquisition. **Mina Mazari:** Investigation; formal analysis; data curation; writing – review and editing. **Stephanie Zopp:** Investigation. **Martha Merrow:** Conceptualization; writing – review and editing; funding acquisition. **Nicole Reisch:** Funding acquisition; writing – review and editing. **Luis Gustavo Perez‐Rivas:** Validation; visualization; writing – review and editing; formal analysis; writing – original draft. **Marily Theodoropoulou:** Conceptualization; methodology; data curation; supervision; writing – original draft; writing – review and editing; funding acquisition.

## CONFLICT OF INTEREST STATEMENT

The authors report no conflict of interest.

## ETHICS STATEMENT

The study was approved by the ethics committee of LMU University Hospital (No. 19–0390). All patients gave informed consent.

## Supporting information


**Table S1.** Acrophase and actigraphy counts on work and work‐free days.
**Table S2.** Univariable analysis of MSFsc as determined by MCTQ and actigraphy across the various comorbidities.
**Table S3.** Chronotype in non‐smoking participants with active Cushing's syndrome versus remission versus controls as determined by MCTQ and actigraphy.
**Table S4.** Univariable analysis of MSFsc as determined by MCTQ and actigraphy across the various comorbidities in non‐smoking participants.
**Table S5.** Chronotype in patients in remission with and without glucocorticoid substitution therapy.
**Table S6.** Correlation and agreement between chronotype parameters measured by MCTQ questionnaires and actigraphy (*n* = 29).

## Data Availability

Restrictions apply to the availability of some or all data generated or analysed for this study to preserve patient confidentiality or because they are being used for ongoing studies. The corresponding author will on request detail the restrictions and any conditions under which access to some data may be provided.
