# Supplementary material for: Cushing's syndrome is associated with an advanced chronotype
Source: J Neuroendocrinol. 2026 Oct 8;38(10):e70286. doi: 10.1111/jne.70286 (PMC13649352; doi:10.1111/jne.70286)
Supplement: Supplementary file 1 [file JNE-38-e70286-s001.pdf]

Supplementary Table 1. Acrophase and actigraphy counts on work and work-free days.

Actigraphy, median (IQR)	Active disease (n=13)	Remission (n=13)	Control (n=13)	Overall P-value	Adjusted P-value active vs control	Adjusted P-value active vs remission	Adjusted P-value remission vs control
WD							
Acrophase, h	14.6 (13; 15.2)	14.7 (13.7; 15.8)	14.6 (14.1; 15.7)	0.819			
Alpha counts	208437.1 (147710.1; 233590.2)	176056.4 (150917.4; 225217.2)	219958 (197646.5; 313927.1)	0.133			
Rho counts	22549.1 (7786.5; 33553.2)	18206.2 (6111; 37336.8)	6503 (3433.2; 14619.5)	0.088	0.117	1.00	0.266
Total counts	236577.7 (162692.6; 255426)	207450.9 (155591.9; 252376.9)	231226.3 (215649.1; 318341.7)	0.254			
FD							
Acrophase, h	13.9 (13; 15.1)	14.8 (13.3; 15.8)	15.4 (14.8; 16.7)	0.030	0.028	1.00	0.247
Alpha counts	173281.3 (143169.4; 218657.3)	203518.3 (173029.7; 215478.3)	218833 (190102.8; 281731.5)	0.234			
Rho counts	23825 (16125.2; 35396.5)	16402.7 (5124.7; 30954.7)	4673.3 (2971.9; 15486.8)	0.054	0.070	1.00	0.190
Total counts	205683.7 (172338; 240588)	214266.3 (186305.3; 272199.1)	240656.8 (208812.8; 317130.7)	0.153			

Data were analysed with Kruskal-Wallis test adjusted for multiple comparisons. Adjusted p-values for multiple comparisons are shown for comparisons with overall p-value < 0.1.
WD, workdays; FD, work-free days.

Supplementary Table 2. Univariable analysis of MSFsc as determined by MCTQ and actigraphy across the various comorbidities.

	MCTQ			Actigraphy		
	N	Spearman's rho	p-value	N	Pearson correlation	p-value
Age, years	119	-0.141	0.127	39	0.034	0.835
BMI, kg/m^2	119	-0.046	0.620	39	0.086	0.603
		Median (IQR)	p-value		Mean (SD)	p-value
Sex	119			39		
Female		3.1 (2.5; 4.1)	0.182		3.7 (1.1)	0.321
Male		3.6 (2.5; 5.2)			4.5 (0.7)	
BMI	119		0.382	39		0.794
<25		3 (2.4; 3.8)			3.7 (0.8)	
25-29.9		3.4 (2.5; 4.3)			3.9 (1.5)	
≥30		3.2 (2.4; 5.1)			4.1 (0.9)	
Menopause	83		0.955			0.358
No		3.3 (2.5–4.0)			3.7 (1.1)	
Yes		3.0 (2.6–4.2)			4.1 (1.0)	
Smoker	119		0.526	39		0.800
No		3.3 (2.5; 4.3)			3.9 (1.1)	
Yes		2.9 (2.2; 4.0)			3.4 (0.9)	
**Comorbidities**						
Diabetes	99		0.569	31		0.392
No		3.2 (2.5; 4.3)			3.8 (1.1)	
Yes		2.9 (2.1; 4.5)			3.6 (1.0)	
Metabolic syndrome	99		0.998	30		0.533
No		3.2 (2.5; 4.2)			3.8 (1.1)	
Yes		3.3 (2.2; 5.2)			3.5 (1.1)	
Hypercholesterolemia	99		0.594	30		0.372
No		3.3 (2.5; 4.3)			3.9 (1.2)	
Yes		3 (2.2; 4.3)			3.6 (1.0)	
Hypertriglyceridemia	99		0.797	30		0.262
No		3.1 (2.5; 4.3)			3.9 (1.1)	
Yes		3.4 (2.3; 4.8)			3.3 (1.1)	
Mixed hyperlipidaemia	99		0.289	30		0.145
No		3.2 (2.5; 4.3)			3.9 (1.1)	
Yes		3.0 (2.0; 3.7)			3.1 (1.1)	
Hypertension	99		0.224	31		0.621
No		3.3 (2.7; 4.3)			3.9 (1.8)	
Yes		3.0 (2.3; 3.9)			3.7 (1.0)	
Arteriosclerosis	99		0.192	31		-
No		3.2 (2.5; 4.3)			3.8 (1.1)	
Yes		4.4 (2.9; 5.5)			-	
Cardiovascular events	99		0.713	31		0.799
No		3.2 (2.4; 4.3)			3.8 (1.1)	
Yes		3.2 (2.8; 4.2)			4.0 (0.8)	
Sleep apnoea	99		0.954	31		-
No		3.2 (2.5; 4.3)			3.8 (1.1)	
Yes		3.6 (2.5; 3.9)			-	
Psychiatric symptoms	99		0.014	30		0.076
No		3.4 (2.8; 4.4)			4.0 (1.1)	
Yes		2.6 (2.2; 4.4)			3.1 (0.8)	
Sleep disturbance	99		0.016	30		0.133
No		3.4 (2.7; 4.4)			4.0 (1.2)	
Yes		2.8 (1.8; 3.5)			3.3 (0.9)	

Mann-Whitney U test was used for MCTQ data except BMI, for which we used. Kruskal-Wallis test.
Student's t-test was used for actigraphy data except BMI, for which we used ANOVA.
MCTQ, Munich chronotype questionnaire.

Supplementary Table 3. Chronotype in non-smoking participants with active Cushing's syndrome vs. remission vs. controls as determined by MCTQ and actigraphy.

	Active disease	Remission	Controls	p-value active vs control	p-value active vs remission	p-value remission vs. control
**MCTQ*** Median (IQR)	n=15	n=55	n=34			
MSFsc	2.2 (1.1; 3.1)	3.3 (2.6; 4.3)	3.7 (3.2; 4.0)	<0.001	0.005	0.451
MSF	2.6 (1.0; 3.1)	3.6 (2.9; 4.3)	3.9 (3.5; 4.2)	0.001	0.005	0.967
Weekly sleep duration	6.7 (4.9; 7.2)	7.4 (6.6; 8.1)	7.3 (6.5; 7.8)	0.278	0.048	1.000
Sleep Onset WD	22.8 (22.0; 23.3)	23.2 (22.6; 24.0)	23.6 (23.1; 24.2)	0.015	0.345	0.177
Sleep Offset WD	5.0 (4.0; 6.0)	6.0 (5.8; 7.0)	6.5 (6.3; 7.4)	<0.001	0.003	0.223
Sleep duration WD	6.2 (5.0; 7.1)	7.3 (6.4; 7.9)	7.1 (6.7; 7.6)	0.116	0.021	1.000
Sleep Onset FD	23.2 (22.0; 23.8)	23.9 (23.2; 24.5)	24.1 (23.5; 24.5)	0.018	0.121	0.742
Sleep Offset FD	5.0 (4.2; 6.5)	7.5 (6.6; 8.2)	8.0 (7.0; 8.4)	0.002	0.010	1.000
Sleep duration FD	6.8 (4.7; 7.6)	7.8 (6.8; 8.9)	7.8 (7.0; 8.2)	0.282	0.126	1.000
**Actigraphy#** mean (SD)	n=9	n=13	n=10			
MSFsc	3.2 (1.1)	4.0 (1.0)	4.4 (0.8)	0.030	0.126	0.667
MSF	3.2 (1.1)	4.0 (1.0)	4.5 (0.9)	0.015	0.178	0.354
Weekly sleep duration	7.9 (0.8)	8.1 (0.7)	7.9 (0.9)	0.996	0.908	0.938
Sleep Onset WD	22.5 (0.9)	23.6 (1.7)	23.8 (1.0)	0.098	0.186	0.879
Sleep Offset WD	6.5 (1.0)	7.7 (1.5)	7.6 (1.0)	0.116	0.078	0.997
Sleep duration WD	7.9 (0.8)	8.1 (0.9)	7.8 (0.8)	0.933	0.885	0.661
Sleep Onset FD	23.2 (1.1)	24.0 (1.2)	24.4 (1.0)	0.067	0.255	0.656
Sleep Offset FD	7.1 (1.6)	7.9 (0.9)	8.7 (1.2)	0.021	0.280	0.303
Sleep duration FD	7.8 (1.5)	7.9 (0.8)	8.3 (1.3)	0.679	0.988	0.724

*Kruskal-Wallis test corrected for multiple comparisons. #ANOVA corrected using Tukey's adjustment for multiple comparisons. MSF and MSFsc data are expressed in decimal time.
MCTQ, Munich chronotype questionnaire; MSFsc, mid-sleep on free days corrected for sleep debt; MSF, mid-sleep on free days; WD, workdays; FD, (work) free days.

Supplementary Table 4. Univariable analysis of MSFsc as determined by MCTQ and actigraphy across the various comorbidities in non-smoking participants.

	MCTQ			Actigraphy		
	N	Spearman's rho	p-value	N	Pearson correlation	p-value
Age, years	104	-0.202	0.040	32	0.196	0.283
BMI, kg/m^2	104	-0.017	0.868	32	0.068	0.721
		Median (IQR)	p-value		Mean (SD)	P-value
Sex	104			32		
Female		3.3 (2.6–4.0)	0.252		3.8 (1.1)	0.318
Male		3.5 (2.7–4.6)			4.2 (0.6)	
BMI	104		0.929	32		0.857
<25		3.4 (2.8–3.9)			3.8 (0.7)	
25-29.9		3.3 (2.7–4.2)			4.0 (1.5)	
≥30		3.2 (2.5–4.8)			4.0 (0.9)	
Menopause	70		0.91	26		0.019
No		3.3 (2.5–4.1)			3.6 (1.1)	
Yes		3.2 (2.6–4.0)			4.6 (0.7)	
**Comorbidities**						
Diabetes	84		0.53	25		0.778
No		3.3 (2.5–4.3)			3.9 (1.2)	
Yes		2.9 (2.5–4.0)			3.6 (1.0)	
Metabolic syndrome	84		0.89	25		0.631
No		3.3 (2.5–4.3)			3.9 (1.2)	
Yes		3.3 (2.4–5.2)			3.6 (1.2)	
Hypercholesterolemia	84		0.873	25		0.838
No		3.2 (2.5–4.3)			3.9 (1.2)	
Yes		3.4 (2.2–4.2)			3.8 (1.1)	
Hypertriglyceridemia	84		0.922	25		0.372
No		3.2 (2.6–4.3)			4.0 (1.1)	
Yes		3.4 (2.3–4.6)			3.4 (1.3)	
Mixed hyperlipidaemia	84		0.156	25		0.292
No		3.3 (2.5–4.3)			4.0 (1.1)	
Yes		2.7 (1.7–3.6)			3.1 (1.3)	
Hypertension	84		0.457	25		0.621
No		3.2 (2.6–4.3)			3.8 (1.2)	
Yes		3.3 (2.3–3.9)			3.9 (1.1)	
Arteriosclerosis	84		0.433	25		-
No		3.2 (2.5–4.3)			3.8 (1.2)	
Yes		3.8 (3.2–4.7)			-	
Cardiovascular events	84		0.657	25		0.019
No		3.2 (2.5–4.3)			3.8 (1.2)	
Yes		3.3 (2.8–3.8)			4.4 (0.1)	
Sleep apnoea	84		0.542	25		-
No		3.2 (2.5–4.3)			3.8 (1.1)	
Yes		3.7 (3.4–3.9)			-	
Psychiatric symptoms	84		0.038	25		0.211
No		3.4 (2.8–4.3)			4.0 (1.2)	
Yes		2.6 (2.3–3.4)			3.2 (1.0)	
Sleep disturbance	84		0.027	25		0.484
No		3.4 (2.7–4.3)			3.9 (1.2)	
Yes		2.6 (1.6–3.3)			3.5 (1.1)	

Mann-Whitney U test was used for MCTQ data except BMI, for which we used. Kruskal-Wallis test.
Student's t-test was used for actigraphy data except BMI, for which we used ANOVA.
MCTQ, Munich chronotype questionnaire.

Supplementary Table 5. Chronotype in patients in remission with and without glucocorticoid substitution therapy.

	Remission + GC substitution	Remission no GC substitution	P-value*
**MCTQ,** median (IQR)	n=30	n=31	
MSFsc	3.33 (2.46; 4.18)	3.01 (2.8; 4.40)	0.762
MSF	4.00 (2.79; 4.25)	3.56 (3.02; 4.53)	0.745
Weekly sleep duration	7.49 (6.57; 8.26)	7.38 (6.54; 7.96)	0.415
Sleep Onset WD	23.00 (22.42; 24.50)	23.17 (22.96; 23.98)	0.371
Sleep Offset WD	6.00 (5.58; 7.00)	6.00 (5.69; 7.04)	0.930
Sleep duration WD	7.50 (6.33; 8.08)	7.29 (6.31; 7.84)	0.305
Sleep Onset FD	23.75 (23.17; 24.50)	23.94 (23.17; 24.54)	0.649
Sleep Offset FD	7.50 (6.50; 8.50)	7.00 (6.88; 8.44)	0.954
Sleep duration FD	7.83 (6.75; 9.30)	7.83 (6.73; 8.52)	0.697
**Actigraphy,** mean (SD)	n=6	n=7	p-value#
MSFsc	4.21 (1.16)	3.81 (0.69)	0.468
MSF	4.24 (1.18)	3.62 (0.61)	0.269
Weekly sleep duration	8.01 (0.62)	8.10 (0.94)	0.847
Sleep Onset WD	24.47 (1.74)	22.51 (1.11)	0.038
Sleep Offset WD	8.46 (1.55)	6.76 (0.86)	0.038
Sleep duration WD	7.99 (0.84)	8.25 (1.07)	0.631
Sleep Onset FD	24.21 (1.39)	23.76 (0.88)	0.512
Sleep Offset FD	8.28 (1.07)	7.48 (0.53)	0.125
Sleep duration FD	8.07 (0.78)	7.72 (0.77)	0.435

MSF and MSFsc data are expressed in decimal time.
*Mann-Whitney U test was used for MCTQ data.
#Student´s t-test was used for actigraphy data.
GC, glucocorticoid; MCTQ, Munich chronotype questionnaire; MSF, mid-sleep on free days; MSFsc, mid-sleep on free days corrected for sleep debt; WD, workdays; FD, free days.

Supplementary Table 6. Correlation and agreement between chronotype parameters measured by MCTQ questionnaires and actigraphy (n=29).

	Correlation (Pearson)		Agreement (Bland-Altman analysis)			
	Correlation coefficient	p-value	Systematic bias (mean difference, hours)	Limit of agreement (hours)	Proportional bias (correlation coefficient)	p-value
MSFsc	0.619	0.0003	-0.05	(-2.42; 2.32)	0.508	0.005
MSF	0.669	<0.0001	0.13	(-2.25; 2.51)	0.538	0.003
Weekly sleep duration	0.397	0.033	-1.24	(-4.33; 1.85)	0.673	<0.0001
Working days						
Sleep onset	0.820	<0.0001	0.34	(-1.22; 1.91)	-0.261	0.172
Sleep offset	0.705	<0.0001	-0.95	(-2.96; 1.06)	-0.001	0.996
Sleep duration	0.543	0.002	-1.25	(-3.47; 0.97)	0.458	0.013
Free days						
Sleep onset	0.638	<0.0001	0.50	(-1.97; 2.96)	0.492	0.007
Sleep offset	0.590	0.0007	-0.25	(-3.51; 3.02)	0.535	0.003
Sleep duration	0.409	0.027	-0.74	(-4.04; 2.55)	0.481	0.008

MCTQ, Munich chronotype questionnaire; MSF, mid-sleep on free days; MSFsc, mid-sleep on free days corrected for sleep debt.
